# Aildenafil citrate with superior PDE5 selectivity ameliorates cognitive and behavioral impairments in APP/PS1 Alzheimer’s disease mice: a preclinical phenotypic study

**DOI:** 10.3389/fphar.2026.1933588

**Published:** 2026-09-16

**Authors:** Shaowei Han, Fang Liu, Dawei Wang, Gengshen Song

**Affiliations:** 1 Beijing Youcare Kechuang Pharmaceutical Technology Co., Ltd., Beijing, China; 2 Youcare Pharmaceutical Group Hangzhou Tianlong Pharmaceutical Co.,Ltd., Hangzhou, Zhejiang, China

**Keywords:** aildenafil citrate (AC), Alzheimer’s disease (AD), Aβ deposition, cognitive dysfunction, oxidative stress, phosphodiesterase 5 (PDE5)

## Abstract

**Introduction:**

Alzheimer’s disease (AD) imposes a significant global healthcare burden, and current available pharmacotherapies provide limited therapeutic benefits. Disrupted phosphodiesterase 5 (PDE5) signaling has been implicated in the pathogenesis of AD and other neurodegenerative disorders, positioning PDE5 inhibitors (PDE5Is) as a promising therapeutic strategy for AD treatment. This study evaluated the preclinical therapeutic potential of aildenafil citrate (AC), a selective PDE5I approved in China for the treatment of erectile dysfunction (ED), in AD treatment.

**Methods:**

Molecular docking, molecular dynamics (MD) simulations, and MM-GBSA binding free energy calculations were integrated to explore the molecular mechanisms of aildenafil. The APP/PS1 transgenic mouse model of AD was employed to assess the in vivo efficacy of AC through behavioral, pathological, and oxidative assessments.

**Results:**

In silico analyses indicated that aildenafil exhibited a higher predicted affinity through a strengthened electrostatic network anchored by key bidentate hydrogen bonds with Gln817 and enhanced π‐π stacking interactions with Phe820. Behavioral assessments, including the Barnes maze and Open field tests, demonstrated that AC significantly ameliorates cognitive dysfunction and anxiety-like impairments. Pathological examinations revealed improved hippocampal morphology, upregulated neprilysin (NEP) expression, and reduced Aβ accumulation. The decreased malondialdehyde (MDA) levels further suggested a potential attenuation of systemic oxidative stress and provided preliminary support for further investigation into its effects on central redox status.

**Conclusion:**

These findings offer an initial phenotypic characterization of AC in an AD mouse model, underscoring the need for comprehensive mechanistic studies to fully evaluate its potential as a repurposed AD therapeutic candidate.

## Graphical Abstract

**Figure:**
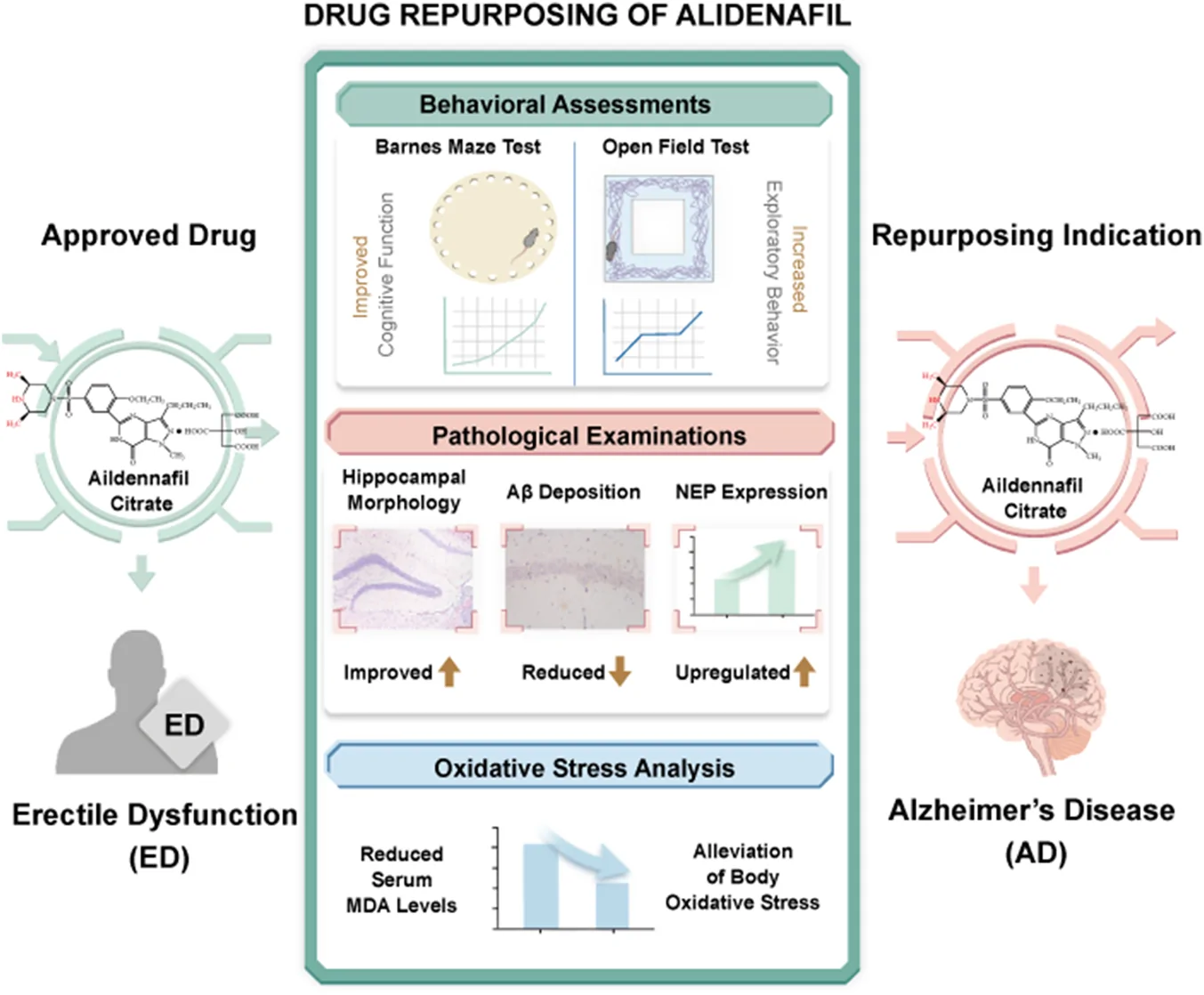

## 1 Introduction

Alzheimer’s disease (AD) is a progressive degenerative disorder characterized by memory impairment and dementia (Alzheimer’s Association, 2015). As the most common cause of dementia, AD represents the fifth leading cause of death among individuals aged 65 and older, affecting over 7 million people in the United States (Reitz and Mayeux, 2014; Alzheimer’s Association, 2025). The prevalence of AD continues to rise with the globally aging populations. Projections suggest a near fourfold increase in AD cases by 2050, which would dramatically escalate the global burden (Yu et al., 2025).

Current standard interventions for AD are limited to cholinesterase inhibitors and N-methyl-D-aspartate (NMDA) receptor antagonists (Atri, 2019). Although widely used, these treatments offer only modest symptomatic relief, prompting the development of novel therapeutic approaches. To date, three monoclonal antibodies (mAbs), aducanumab, donanemab, and lecanemab, have been approved for AD treatment (Cummings et al., 2024; Zhang et al., 2024). These therapeutics target amyloid-β (Aβ), a key molecular driver of Alzheimer’s pathogenesis and progression. However, their exceptionally high cost has raised concerns about cost-effectiveness, even in developed countries (Ross et al., 2022; Xiong et al., 2025; Nguyen et al., 2024). Furthermore, clinical trials into these agents have reported inconsistent therapeutic outcomes, highlighting unmet demand for more effective, affordable, and safer treatment alternatives (Avgerinos et al., 2024).

Emerging evidence implicates disrupted cyclic guanosine monophosphate (cGMP) signaling in the pathogenesis of neurodegenerative diseases, including AD (Jehle and Garaschuk, 2022; Tropea et al., 2022). Phosphodiesterase 5 (PDE5), a member of the phosphodiesterase (PDE) family, selectively hydrolyzes cGMP to 5′-GMP, thereby tightly regulating intracellular cGMP concentrations (Ahmed et al., 2021). PDE5 is predominantly expressed in the smooth muscle of the corpus cavernosum and highly enriched in brain regions including the substantia nigra, cerebellum, caudate nucleus, and hippocampus. Notably, PDE5 levels are significantly elevated in the temporal cortex of AD patients compared with age-matched healthy control individuals, supporting PDE5 inhibition as a potential therapeutic strategy against AD by restoring cGMP signaling within the central nervous system (Puzzo et al., 2005; Kang et al., 2022). By blocking cGMP hydrolysis to 5′-GMP, PDE5 inhibitors activate downstream signaling pathways involving cAMP-responsive element binding protein (CREB), SIRT1, and Nuclear factor erythroid-2-related factor 2 (Nrf2) signaling and the suppression of NF-κB and GSK-β signaling (Wu et al., 2018; Liu et al., 2019; Zuccarello et al., 2020; Singh et al., 2024). Such modulation mitigates key AD pathologies—Aβ aggregation, tau hyperphosphorylation, neuroinflammation, and oxidative stress—and concomitantly enhances cerebrovascular function (Scheltens et al., 2021; Verma et al., 2022; Zheng and Wang, 2025). Drug repurposing studies have successfully identified the potential of sildenafil, the first oral PDE5I approved for ED, as a candidate for alleviating the impaired cognitive functions in preclinical AD models (Devan et al., 2006; Puzzo et al., 2009). Subsequent studies have extended these observations to other PDE5 inhibitors, including tadalafil and vardenafil, which have demonstrated neuroprotective effects in various AD models through modulation of the NO/cGMP/PKG/CREB signaling axis, reduction of neuroinflammation, and attenuation of oxidative stress (Zhang et al., 2013; Zuccarello et al., 2020). A recent meta-analysis of preclinical studies further highlighted the anti-inflammatory and antioxidant actions of PDE5Is, which may help counter neuroinflammation and oxidative stress in neurodegenerative diseases including AD (Uppuluri et al., 2026). Despite these encouraging preclinical findings, no PDE5 inhibitor has yet been approved for AD treatment, and clinical studies assessing cognitive functions using marketed PDE5 inhibitors have not yielded conclusive results. This gap underscores the need for continued investigation into PDE5Is with improved pharmacological profiles—such as enhanced selectivity, potency, and blood–brain barrier penetration—that may offer greater therapeutic potential for AD.

Aildenafil citrate (AC), a novel PDE5I exhibiting superior selectivity and specificity, was approved in China in 2021 for the treatment of ED. Given the emerging role of PDE5 inhibition in neurodegeneration, we aimed to investigate the preclinical efficacy of AC in a transgenic mouse model of AD. Our phenotypic observations in AD model mice suggest that repurposing PDE5Is merits further exploration as a potential therapeutic direction for AD, with comprehensive brain penetration and robust central PDE5 inhibitory activity still required to confirm translational value.

## 2 Materials and methods

### 2.1 Synthesis of AC

AC was synthesized as previously described (Supplementary Scheme S1) (Vendeville et al., 2020). Briefly, 2-ethoxybenzoic acid reacted with chlorosulfonic acid and thionyl chloride to yield compound II, followed by amination with cis-2,6-dimethylpiperazine to produce compound III. Acylation of III afforded acyl chloride IV, which was coupled with compound V (synthesis seen in CN1246478A) using 4-dimethylaminopyridine and trimethylamine to obtain compound VI. Final cyclization of compound VI with potassium *tert*-butoxide yielded the target compound AC (compound I′). Identity was confirmed by ^1^H-NMR and ^13^C-NMR (Supplementary Figures S1 and S2), along with a high purity (>99.5%) determined by HPLC.

### 2.2 Docking analysis

#### 2.2.1 Initial structure preparation

PDE5 (PDB: 1UDT) was prepared by removing water and co-crystallized ligands using PyMOL (Roymans et al., 2017). Hydrogen atoms were added and protonation states of receptor and ligands (sildenafil, aildenafil) were optimized at pH 7.4, with Gasteiger charges assigned using Open Babel (O’Boyle et al., 2011). Ligands were then energy-minimized using MMFF94 (Gasteiger and Marsili, 1980; Trott and Olson, 2010), and all structures converted to PDBQT format for docking.

#### 2.2.2 Molecular docking

The docking box (19.25 × 25.69 × 19.43 Å) was centered on the PDE5 active site at coordinates (1.440, 67.015, 83.530 Å), extending 12 Å beyond the co-crystallized ligand to accommodate flexibility. AutoDock Vina (Eberhardt et al., 2021) was used with an exhaustiveness of 48, and the top 50 poses ranked by predicted binding affinity ( $\Delta G_{bind}$ ), were retained for analysis via the Vina scoring function (Trott and Olson, 2010).

#### 2.2.3 Conformational clustering and analysis

Retained poses (n = 50 per ligand) were clustered using UPGMA (Cichero et al., 2021) with a 1.5 Å heavy-atom root-mean-square deviation (RMSD) cutoff following iterative alignment. Predominant clusters were characterized by population (n, %), mean predicted binding affinity ( $\Delta G_{bind}$ , kcal mol^−1^; mean ± SD), and intra-cluster RMSD (mean ± SD). Differences in binding affinity between sildenafil and aildenafil clusters were assessed using a two-tailed Wilcoxon rank-sum test.

### 2.3 Molecular dynamics (MD) simulations

#### 2.3.1 System preparation

MD simulations were performed using GROMACS 2024.5 (Abraham et al., 2015). The ligand was parameterized with the GAFF2 (Wang et al., 2004) and AM1-BCC (Jakalian et al., 2002) charges via Antechamber (Case et al., 2023), with topologies generated by Sobtop (Zhang et al., 2022). The PDE5 protein was modeled using the AMBER99SB-ILDN (Hornak et al., 2006; Lindorff-Larsen et al., 2010). The complex was solvated in a TIP3P (Jorgensen et al., 1983) dodecahedral box with 1.0 nm clearance, neutralized, and adjusted to 0.15 M NaCl. Energy minimization involved successive steepest descent (F_*max*_ < 500 kJ mol^−1^ nm^−1^) and conjugate gradient (F_*max*_ < 100 kJ mol^−1^ nm^−1^) steps (Abraham et al., 2015).

#### 2.3.2 Equilibration, production, and trajectory analysis

The system was equilibrated via 100-ps NVT and 100-ps NPT stages, applying 1,000 kJ mol^−1^ nm^−2^ heavy-atom restraints. Temperature (310 K) and pressure (1.0 bar) were regulated by the V-rescale thermostat (Bussi et al., 2007) and C-rescale barostat (Bernetti and Bussi, 2020). Subsequently, a 100-ns production MD was performed in the NPT ensemble (2-fs time step) using the Parrinello-Rahman barostat (Zhu et al., 2017). LINCS (Hess et al., 1997) constrained hydrogen-containing bonds. For all stages, PME (Darden et al., 1993) handled long-range electrostatics, with a 1.0 nm cutoff for non-bonded interactions. Trajectories were periodic boundary conditions (PBC)-corrected and centered. Root-mean-square deviation (RMSD), root-mean-square Fluctuation (RMSF), and hydrogen bond frequency were analyzed using GROMACS.

#### 2.3.3 Molecular mechanics generalized born surface area (MM-GBSA) analysis

Binding free energies and per-residue decomposition were calculated using gmx_MMPBSA (Valdés-Tresanco et al., 2021) to quantify inhibitor affinity. Analysis was performed on the final 40 ns of the production trajectory.

### 2.4 *In vitro* inhibitory activity

The expression and enzyme assays of PDE isoforms were performed as reported (Weeks et al., 2005; Wu et al., 2017). Briefly, recombinant plasmids were transformed into *E. coli* BL21 (Codonplus), cultured in LB medium at 37 °C to an A600 of 0.6–0.8, and induced with 0.1 mM IPTG at 15 °C for 24 h. Recombinant proteins were purified via Ni-NTA, Q, and Superdex 100 chromatography. PDE activities were determined using cGMP or cAMP as substrates. Radiolabeled [8-^3^H]-cAMP and [8-^3^H]-cGMP were diluted 100-fold for subsequent assays. For PDE5A detection, the reaction mixture contained substrate mixture, test compound or DMSO, and diluted enzyme, with incubation at room temperature for 15 min. Enzyme-free buffer served as control. Reactions were terminated by ZnSO_4_ and Ba(OH)_2_, and supernatant radioactivity was quantified via liquid scintillation counting. Each compound (7–9 concentrations) was tested in at least three independent replicates. Inhibition rates were calculated and IC_50_ values were determined by nonlinear regression using GraphPad Prism software.

### 2.5 Ethics and animals

All animal procedures were conducted in accordance with the Guide for the Care and Use of Laboratory Animals (Cockerill et al., 2021) and were approved by the Institutional Animal Care and Use Committee of Jiangxi ZhongHong BoYuan Biological Technology Co., Ltd. (no. 2022020901). This study also adhered to the ARRIVE guidelines for reporting animal research.

Both male and female APPswe/PS1dE9 transgenic (APP/PS1) mice and their wild-type (WT) littermates were purchased from SPF (Beijing) biotechnology Co., Ltd. All animals were housed in SPF-grade facilities at Jiangxi ZhongHongBoYuan Biological Technology Co., Ltd., with a 12-h light/dark cycle (20 °C–26 °C) and free access to food and water until 7 months of age. Following the completion of all behavioral assessments, mice were anesthetized by intraperitoneal injection of 1% pentobarbital solution at 50 mg/kg. Blood samples were collected immediately, followed by euthanasia via cervical dislocation and subsequent tissue harvest.

### 2.6 Experimental design

Prior to dosing initiation, a Barnes maze test was conducted to establish baseline performance. APP/PS1 mice were randomly assigned to five groups using a computer-generated randomization sequence (stratified by Barnes maze escape latency), ensuring comparable cognitive performance across groups at study onset (Supplementary Figure S3). All behavioral tests were conducted by experimenters blinded to group allocation; video recordings were analyzed by a separate investigator who was also blinded to treatment groups. Histopathological and biochemical analyses were performed by technicians blinded to group assignments. The blinding code was broken only after all data analyses were completed.

The five groups consisted of the AD model control (MC, 0.9% saline), positive control (PC, 2.5 mg/kg memantine hydrochloride), low-dose AC (AC1, 5 mg/kg), mid-dose AC (AC2, 10 mg/kg) and high-dose AC (AC3, 15 mg/kg) groups (n = 6, male: female = 3:3). Age-matched WT C57BL/6 mice served as the negative control (NC). All groups except the NC group were dosed once daily for 5 weeks via sexoral gavage.

The AC doses (5, 10, and 15 mg/kg) were selected to span the predicted therapeutic range based on allometric scaling from human pharmacokinetic studies (30–90 mg in humans) and previous preclinical studies with PDE5 inhibitors in APP/PS1 mice (Puzzo et al., 2009; Pifarre et al., 2011; García-Barroso et al., 2013; Zhu et al., 2015); The 5-week treatment duration was chosen to allow sufficient time for drug accumulation and pathological modulation; Memantine at 2.5 mg/kg was selected based on published protocols demonstrating cognitive improvement in APP/PS1 mice without confounding effects on locomotor activity (Van Dam et al., 2005; Stepanyan et al., 2008; Qiao et al., 2021; Shuvaev et al., 2022).

Barnes maze test and open field test were performed at the end of the treatment period. Subsequently, all animals were euthanized for blood collection and histological analysis.

### 2.7 Behavioral tests

#### 2.7.1 Barnes maze test

The Barnes maze test was conducted both before treatment initiation and 5 weeks after the completion of drug administration. The apparatus consisted of an elevated circular platform (100 cm in diameter) featuring 20 evenly spaced holes (5 cm in diameter) along its perimeter. One hole was equipped with an escape tunnel (19 cm × 8 cm × 7 cm), others were blank. Four distinct visual cues surrounded the maze for spatial orientation. Bright light and strong wind were applied as aversive stimuli to motivate escape.

On Day 28, each mouse was placed in the maze center for free exploratory habituation. Mice unable to enter the escape tunnel within 3 min were gently guided by the experimenter. From Day 29–34, mice received a 5-min daily training trial. A 15-min interval was arranged between individual tests, during which the maze was thoroughly cleaned with 75% ethanol and all cues were reset to eliminate olfactory traces and ensure environmental consistency. At the start of each trial, mice were confined in a central dark start chamber for 10 s before free exploration. After each trial, the mouse remained in the escape box for 1 min. Individuals failing to locate the tunnel within 240 s were manually guided. On Day 35, a 4-min probe trial was performed with the escape box removed. All sessions were video-recorded and analyzed with tracking software.

#### 2.7.2 Open field test

The Open field test was carried out 1 day after completion of the Barnes maze test. Each mouse was placed individually in the center of a square arena and allowed to explore freely for 5 min. The following behavioral parameters were quantified: total distance, average speed, central distance, time in center, frequency of center entries and activity time in center. The arena was thoroughly cleaned with 75% ethanol between successive trials to eliminate olfactory cues and residual debris.

### 2.8 Histopathological analysis

#### 2.8.1 Hematoxylin and eosin (HE) staining

Mouse hippocampal tissues were fixed in 4% paraformaldehyde, rinsed, and dehydrated in graded ethanol. Tissues were treated with ethanol/xylene and pure xylene, infiltrated with xylene-paraffin mixture and pure paraffin, and embedded in paraffin. After sectioning, baking, deparaffinization, and rehydration, sections were stained with hematoxylin (AR11800-1, BOSTER), differentiated in acid alcohol, blued in Scott’s tap water (G1865, Solarbio), and counterstained with eosin (AR11800-2, BOSTER). Finally, sections were dehydrated, cleared, mounted, and visualized via light microscopy.

#### 2.8.2 Immunohistochemistry

Sections were baked, deparaffinized, and rehydrated. Antigen retrieval was performed using 10 mM citrate sodium buffer (pH 6.0) at 85 °C–95 °C for 10 min. After cooling and washing, 3% hydrogen peroxide was applied to quench endogenous peroxidase. Sections were blocked with 5% BSA and incubated with primary antibodies against NEP or Aβ_42_ (both 1:100). After PBS washing, sections were incubated with HRP-conjugated goat anti-rabbit secondary antibody and visualized with DAB (ZSGB-BIO, Beijing). All slides were counterstained with hematoxylin, dehydrated in graded ethanol, cleared in xylene, and mounted. Images were captured with an Olympus BX43 microscope and analyzed using the MCID computer-based imaging system. Results were quantified as integrated optical density (IOD) per area.

### 2.9 Enzyme-linked immunosorbent assay (ELISA)

The serum level of malondialdehyde (MDA) was quantified using a commercial MDA ELISA kit (Elabscience, Wuhan, China). Briefly, serum samples were centrifuged and added to the pre-coated antibody plates. Subsequently, HRP-labeled antibody was added. After incubation and washing, chromogenic substrates (TMB) were applied. The enzyme-catalyzed color reaction was terminated by acid addition, and the optical density was measured at 450 nm using a microplate reader. MDA concentrations were calculated accordingly.

### 2.10 Statistical analysis

Data were presented as mean ± standard deviation (SD) of at least three biological replicates unless otherwise stated. Statistical analyses were performed on Graphpad Prism v9.0 software. For comparisons involving more than two groups, Bartlett’s test was used to assess the equality of variances. One-way ANOVA was performed for data with equal variances (*p* > 0.05). For comparisons between each treatment group and the model control (MC) group, Dunnett’s *post hoc* test was applied when statistical significance was observed (*p* ≤ 0.05). Dunnett’s T3 test was performed for data with unequal variances (*p* ≤ 0.05).

Effect size was reported as Cohen’s d for pairwise comparisons from Dunnett’s *post hoc* tests, using the pooled standard deviation derived from the ANOVA residual mean square error (√MS_Residual).

For the Barnes maze test (Day 35), escape latency was analyzed using one-way ANOVA, as this endpoint represents a single time-point measurement. The pre-treatment baseline measurement (Day 0) was used exclusively for stratified randomization to ensure comparable cognitive performance across groups prior to drug administration, and was not included in the final statistical comparison. For other single-endpoint measures (e.g., open field, serum MDA), one-way ANOVA was applied as described above. A *p*-value <0.05 was considered statistically significant.

## 3 Results

### 3.1 *In silico* characterization of aildenafil as a potential inhibitor of PDE5

Structurally, AC features a secondary amine (-NH-) and sildenafil contains a tertiary amine (Figure 1). The secondary amine in AC is less basic, which weakens its interactions with organic acids, resulting in lower water solubility but enhanced lipophilicity. Additional differences lie in the position and spatial arrangement of the methyl groups. The methyl group near sildenafil’s tertiary amine acts as an electron-donating substituent to increase amine basicity and water solubility. In AC, two spatially separated methyl groups produce a weaker electron-donating inductive effect, further lowering basicity and aqueous solubility relative to sildenafil.

**FIGURE 1:**
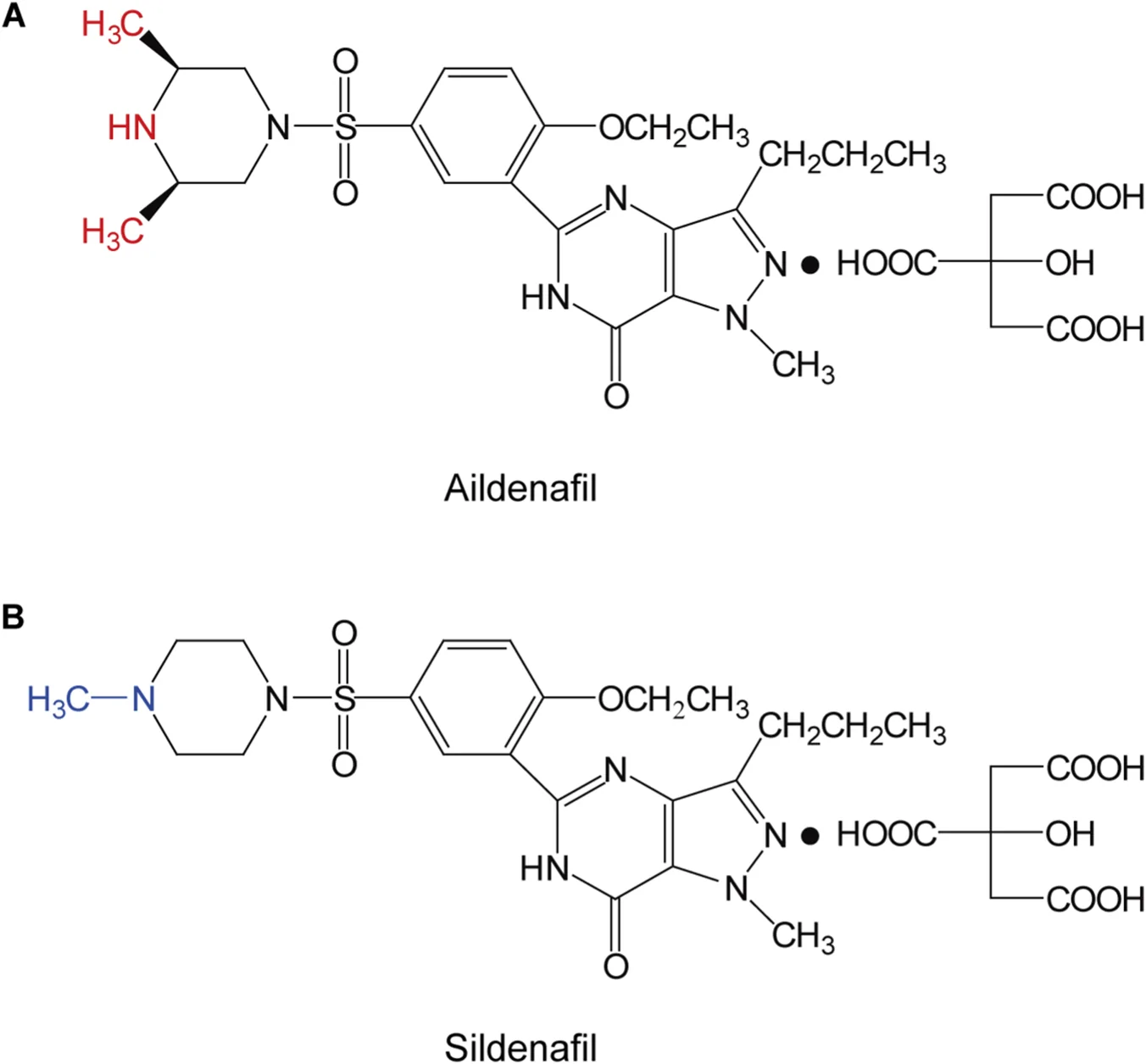
Structure of **(A)** aildenafil and **(B)** sildenafil.

Molecular docking revealed that aildenafil and sildenafil adopted similar binding orientations within the PDE5 catalytic site (Figure 2A). Specifically, the pyrazolopyrimidinone core of aildenafil is anchored in a hydrophobic cavity (Q pocket) formed by Gln817, Phe820, Val782 and Tyr612, forming a π–π stacking interaction with Phe820. Concurrently, the pyrimidinone ring’s C=O and N-H groups form bidentate hydrogen bonds with Gln 817. Within the bimetallic center, His617, Asp 654, Asp764, and His653 coordinate with Zn^2+^, while Asp654 also coordinates with Mg^2+^; this cluster stabilizes the binding site, ensuring precise inhibitor accommodation. Conformational clustering showed that 54% of aildenafil poses converged into a dominant cluster with a mean binding affinity of −8.929 kcal/mol, significantly outperforming sildenafil​ (−8.327 kcal/mol; *p* < 0.05, Wilcoxon rank-sum test) (Table 1; Figure 2B).

**FIGURE 2:**
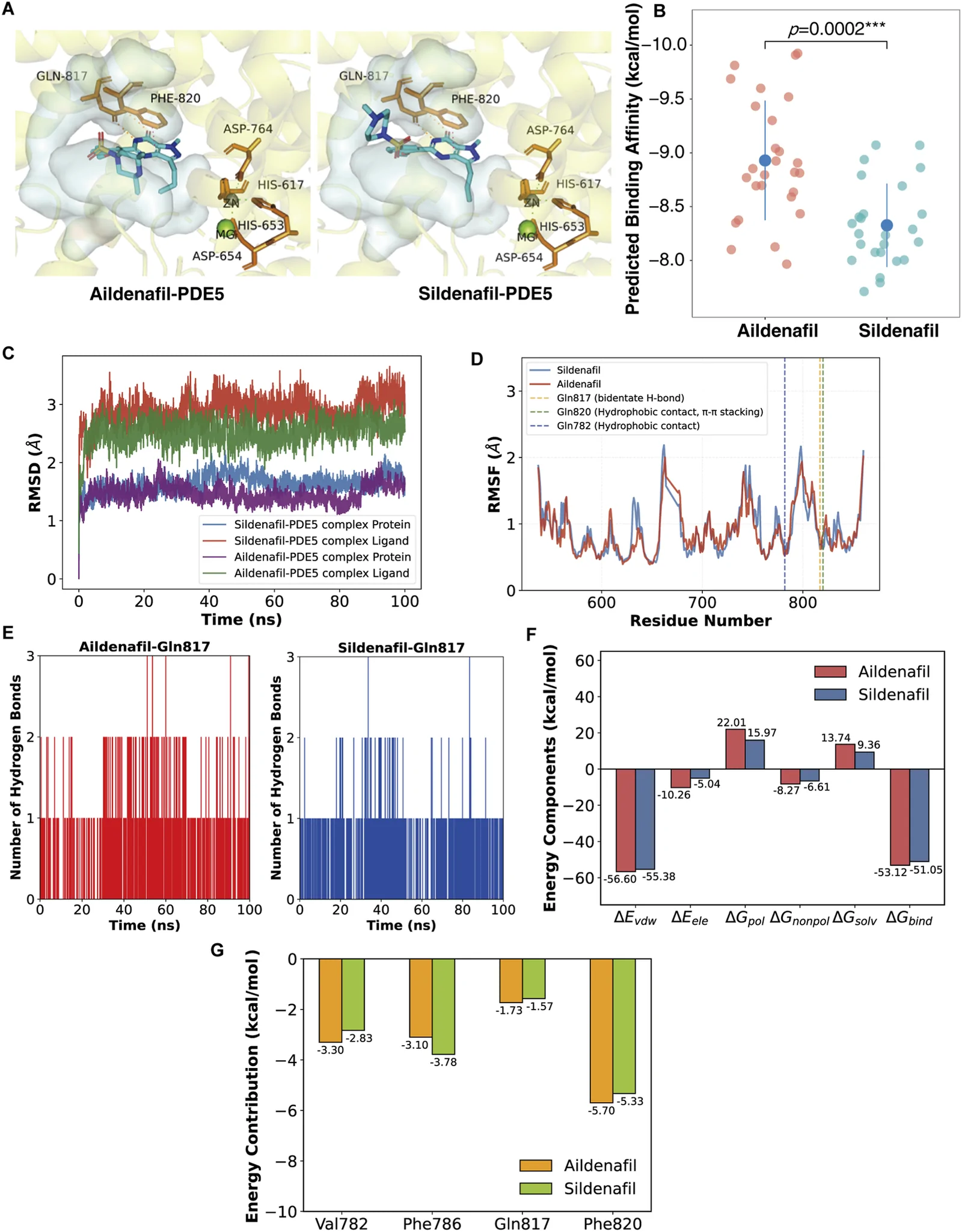
Molecular docking and MD simulations of Aildenafil-PDE5 and Sildenafil-PDE5 complexes. **(A)** Optimal docking poses of aildenafil and sildenafil with PDE5. Key molecular interactions are highlighted: hydrogen bonds (orange), π-π stacking (pink), and metal coordination (green) are represented as dashed lines. Hydrophobic pockets are shown as semitransparent palecyan surfaces. **(B)** Comparison of predicted binding affinities in the dominant cluster for aildenafil and sildenafil with PDE5. **(C)** RMSD of the aildenafil-PDE5 and sildenafil-PDE5 complexes during 100 ns MD simulation. **(D)** RMSF of PDE5 residues in the aildenafil-PDE5 and sildenafil-PDE5 complexes during 100 ns MD simulation. **(E)** Number of hydrogen bonds between Gln817 and the inhibitors in the aildenafil-PDE5 and sildenafil-PDE5 complexes during 100 ns MD simulation. **(F)** MM-GBSA binding free energy components for the aildenafil-PDE5 and sildenafil-PDE5 complexes. **(G)** MM-GBSA per-residue contribution of the key PDE5 residues to the binding free energy in the aildenafil-PDE5 and sildenafil-PDE5 complexes.

**TABLE 1:** Molecular docking results of PDE5 inhibitors.

Ligand	Binding affinity​​ of the optimal conformation (kcal/mol)	Average binding affinity of dominant cluster (kcal/mol)	Average RMSD of dominant cluster (Å)	Proportion of dominant cluster
Sildenafil	−9.840	−8.327	1.005	25/49
Aildenafil	−9.924	−8.929	0.869	27/50

To evaluate the dynamic stability of the docked binding modes, 100 ns MD simulations were performed. RMSD analysis indicated that both aildenafil and sildenafil complexes achieved rapid equilibrium (Figure 2C). Notably, the aildenafil complex exhibited lower backbone and the ligand RMSD values than the sildenafil complex, suggesting that aildenafil forms a more stable binding configuration with PDE5. RMSF analysis showed​ that the key binding regions in both complexes exhibited minimal conformational fluctuations (RMSF < 1.0 Å) (Figure 2D), suggesting that both inhibitors effectively stabilize the active site through a cooperative network of hydrogen bonding, hydrophobic interactions, and π-π stacking. Furthermore, hydrogen bond trajectory analysis revealed that while both ligands maintained pivotal bidentate hydrogen bonds with Gln817, aildenafil exhibited a slightly higher occupancy throughout the simulation (Figure 2E). To quantify binding strength, MM-GBSA analysis was performed. Aildenafil exhibited a higher predicted binding affinity ( $\Delta G_{bind}$ = −53.12 kcal/mol) than sildenafil ( $\Delta G_{bind}$ = −51.05 kcal/mol) (Figure 2F). Per-component energy decomposition revealed that aildenafil possessed approximately twofold enhanced electrostatic interactions compared to sildenafil. Although this incurred to a higher desolvation penalty, the strengthened electrostatic attraction, coupled with optimized hydrophobic packing, synergistically compensated for the polarity-associated energy loss, leading to a lower overall binding free energy. Residue-based energy decomposition further highlighted that aildenafil benefited from more favorable energetic contributions at key residues Val782, Gln817, and Phe820, emerging as the primary stabilizer for the complex (Figure 2G).

To experimentally validate the inhibitory potential suggested by the docking study, we determined the half-maximal inhibitory concentrations (IC_50_) of AC and sildenafil against recombinant human PDE5A. Results showed that AC exhibited potent inhibition with an IC_50_ of 0.66 ± 0.15 nM, which was approximately two-fold lower than that of sildenafil citrate (1.3 ± 0.2 nM) (Table 2). This result confirms that AC is a highly potent PDE5 inhibitor.

**TABLE 2:** Selectivity and specificity of PDE5Is.

PDE subtype	Aildenafil citrate	Sildenafil citrate
PDE1B	>1 μM	>1 μM
PDE2A	>1 μM	>1 μM
PDE3A	>1 μM	>1 μM
PDE4D	>1 μM	>1 μM
PDE5A	0.66 ± 0.15 nM	1.3 ± 0.2 nM
PDE6C	9.1 ± 0.5 nM	17.0 ± 1 nM
PDE7A	>1 μM	>1 μM
PDE8A	>1 μM	>1 μM
PDE9A	>1 μM	>1 μM
PDE10A	>1 μM	>1 μM

### 3.2 AC ameliorates AD-associated cognitive dysfunction and modulates locomotor activity and exploratory behavior in APP/PS1 transgenic mice

To evaluate the effect of AC on spatial learning and memory, we employed the APP/PS1 transgenic mouse model (Figure 3A), a well-established AD model expressing mutated human amyloid precursor protein (APPsw) and presenilin 1 (m146L) (Sasaguri et al., 2017). Compared with the NC group, the MC group exhibited increased latency in entering the target hole, indicating that cognitively impaired AD mice required more time to locate it (Figure 3B, *p* < 0.0001, Cohen’s d = 3.42). Notably, all AC-treated groups (AC1, AC2 and AC3) showed significant dose-dependent reductions in escape latency compared to the MC group (*p* < 0.01 for AC1, Cohen’s d = 1.97; *p* < 0.01 for AC2, Cohen’s d = 2.28; *p* < 0.0001 for AC3, Cohen’s d = 2.74). These reductions corresponded to 31.2%, 35.9%, and 43.2% decreases relative to the model control, demonstrating AC’s efficacy in improving spatial learning and memory upon treatment. Similar improvement was also observed in the PC group administered 2.5 mg/kg memantine hydrochloride (*p* < 0.01, Cohen’s d = 2.37), an FDA-approved medication for the management of moderate to severe AD (Folch et al., 2018).

**FIGURE 3:**
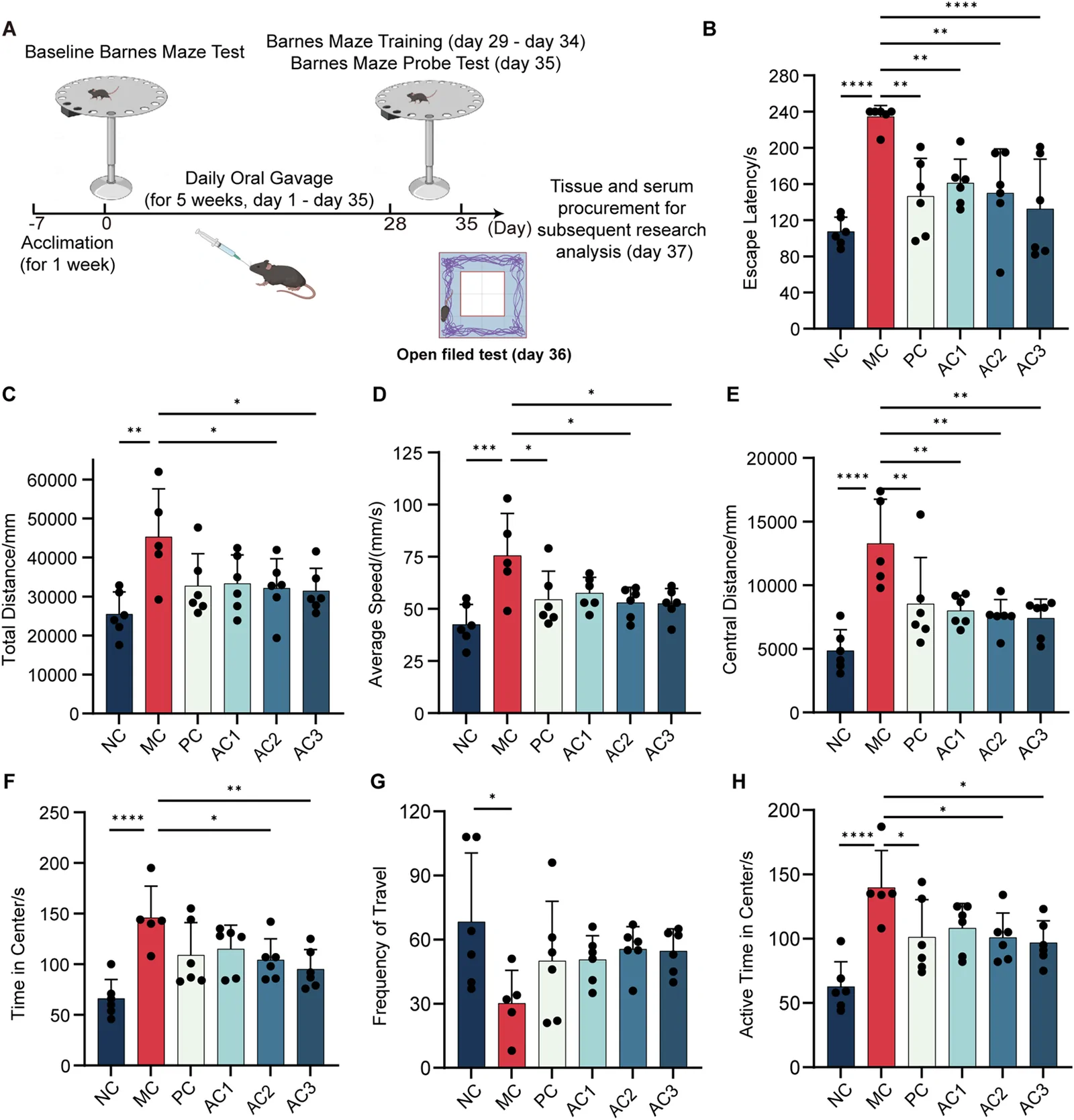
AC improved behavioral functions of APP/PS1 mice in Barnes maze test and open field test. **(A)** Schematic diagram of the experimental design. **(B)** Escape latency in APP/PS1 transgenic mice or WT controls was evaluated by Barnes maze test after treatment. APP/PS1 mice were allowed to freely move in the open field test (OF) arena for 180 s. **(C)** Total distance traveled. **(D)** Average speed. **(E)** Central Distance traveled. **(F)** Time spent in the center. **(G)** Frequency of entry into the center. **(H)** Active time spent in the center. Data were presented as mean ± SD (n = 6 each group). Statistical differences were determined using one-way ANOVA; *p < 0.05; **p < 0.01; ***p < 0.001; ****p < 0.0001.

Locomotor activity and exploratory behavior were evaluated using the Open field test (Walsh and Cummins, 1976). Mice were allowed to freely move in the arena for 180 s. As expected, APP/PS1 mice exhibited hyperlocomotion relative to WT controls, reflected by increased total distance traveled and elevated average speed (Figures 3C,D). Mid-/high-dose AC treatment in the APP/PS1 transgenic mice significantly reversed this hyperactivity, decreasing both total distance (Figure 3C; *p* < 0.05 for AC2, Cohen’s d = 1.66; *p* < 0.05 for AC3, Cohen’s d = 1.75) and average speed (Figure 3D; *p* < 0.05 for AC2, Cohen’s d = 1.96; *p* < 0.05 for AC2, Cohen’s d = 2.01), indicative of restored general locomotor function. Alongside hyperlocomotion, APP/PS1 mice showed marked reduction in central-zone avoidance. Absolute central-zone metrics, including central distance traveled (Figure 3E; *p* < 0.0001, Cohen’s d = 3.64), total time spent in the center (Figure 3F; *p* < 0.0001, Cohen’s d = 3.23), and active time in the center (Figure 3H; *p* < 0.0001, Cohen’s d = 3.43), were substantially increased in MC mice. To exclude the possibility that these elevated absolute central-zone parameters merely reflect general hyperlocomotion, we further calculated the normalized percentage of distance traveled in the central zone (central distance/total distance; Supplementary Figure S4). This normalized index was also significantly higher in APP/PS1 mice (*p* = 0.04, Cohen’s d = 1.59), confirming genuine loss of central-zone avoidance rather than a movement-driven artifact. A reduction in travel frequency was also observed in MC mice (Figure 3G; *p* < 0.05, Cohen’s d = 1.89). Mid- and high-dose AC treatment significantly mitigated these behavioral alterations, lowering absolute central distance, total central-zone time, and active central-zone time (*p* < 0.01 and *p* < 0.05, respectively), with efficacy comparable to memantine. Compared with untreated APP/PS1 mice, memantine- and AC-treated groups showed a non-significant trend toward increased travel frequency in the central zone (Figure 3G). Of note, although treatment groups exhibited a numerical decrease in the normalized central-distance percentage relative to MC mice (Supplementary Figure S4), this trend did not reach statistical significance. Further studies with expanded sample sizes are warranted to more robustly validate this potential effect.

In summary, AC treatment normalized hyperlocomotion and ameliorated the disinhibited open-field behavior in APP/PS1 mice, indicating that AC alleviates AD-associated behavioral deficits with an effect comparable to memantine hydrochloride.

### 3.3 AC reduces pathological abnormalities in the hippocampal region *in vivo*

In AD, the hippocampus is among the earliest regions affected by pathological changes and functional impairment, characterized by aggregation of Aβ, neurodegeneration and neuronal loss. Specifically, the HE results revealed an intact tissue structure with no significant neuronal damage in the NC group (Figure 4A). In contrast, the MC group exhibited visible structural abnormalities, including localized neuronal degeneration featuring pyknotic nuclei, necrotic cells, pericellular vacuolation, and significant vascular congestion in the hippocampus. The AC-treated groups and PC group showed a noticeable reduction in necrotic cells, and attenuated vascular congestion.

**FIGURE 4:**
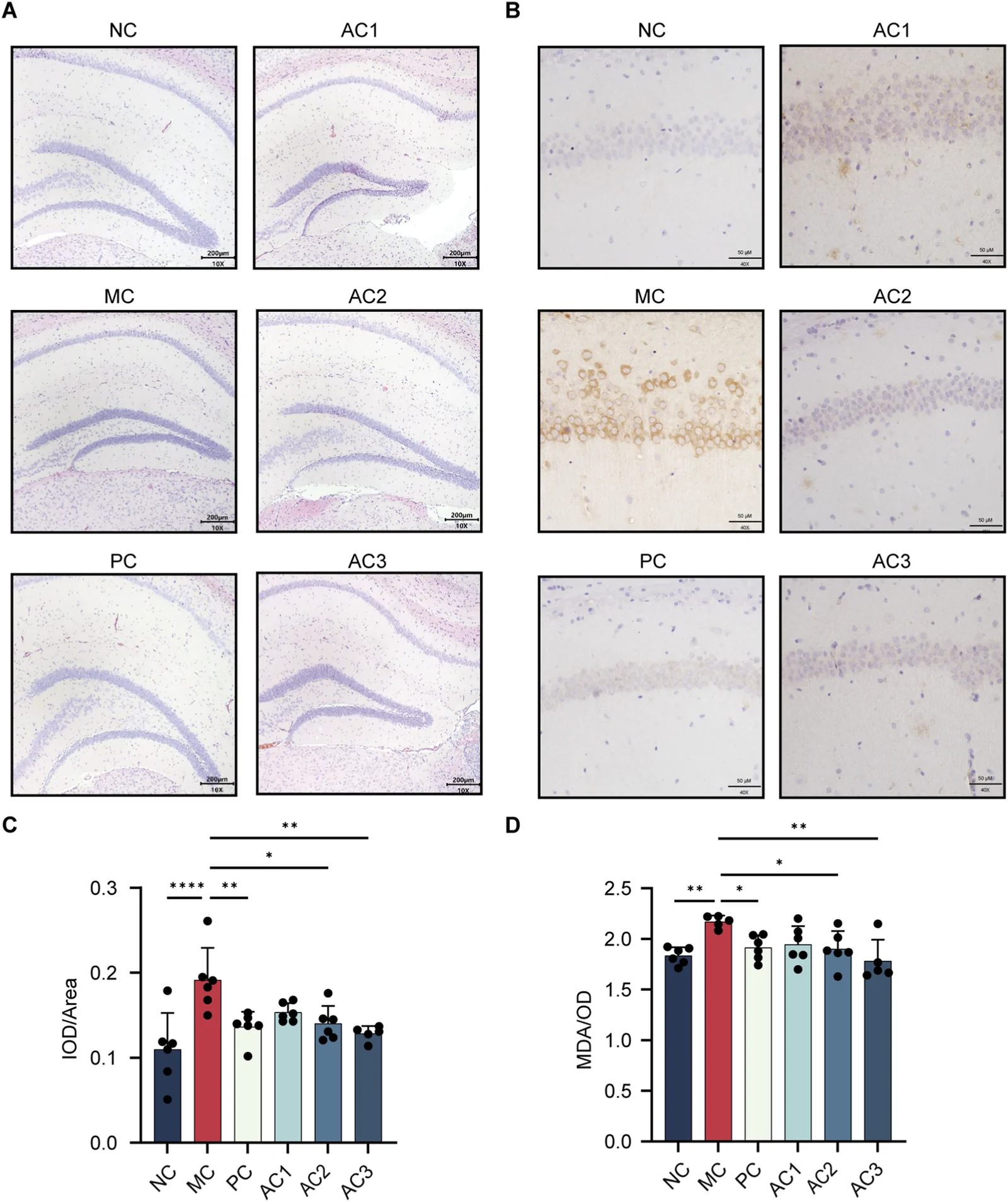
AC reduces pathological abnormalities in the hippocampal region *in vivo*. **(A)** HE staining of the hippocampus tissue of APP/PS1 transgenic mice or WT controls. Representative images of each group are shown. Scale bar = 200 µm. **(B)** Aβ immunohistochemical staining in the hippocampus of APP/PS1 transgenic mice or WT controls. Representative images of each group are shown. Scale bar = 50 µm. **(C)** Quantitative analysis for Aβ staining: IOD per unit area. **(D)** serum MDA level after treatment. Data were presented as mean ± SD (n = 6 each group). Statistical differences were determined using one-way ANOVA; *p < 0.05; **p < 0.01; ****p < 0.0001.

IHC analysis further demonstrated a significant increase in Aβ protein levels in the MC group compared to the NC group. All treatment groups exhibited reduced Aβ accumulation, with high-dose AC showing the most pronounced inhibition of hippocampal Aβ aggregation (Figures 4B,C; *p* < 0.01). Moreover, the expression of NEP, as a key Aβ-degrading enzyme in the hippocampus, was upregulated post AC treatment (Supplementary Figure S5). The mean integrated optical density (IOD/Area) values for NEP in the three AC-treated groups present significant increases of 8.1%, 11.1%, and 13.3% compared to the MC group. Notably, the NEP expression level in the mid-dose AC group was comparable to that of the positive control (memantine), while the high-dose AC group exhibited an even higher level; however, this difference was not statistically significant. These elevations in NEP expression were concomitant with reduced Aβ levels.

Increased cerebral oxidative stress is known to contribute significantly to neuronal degeneration and death in AD (Zhao and Zhao, 2013). Serum MDA, a circulating biomarker reflecting systemic lipid peroxidation, was significantly elevated in the MC group compared with the NC group (Figure 4D; *p* < 0.01, Cohen’s d = 2.26). Both AC and memantine treatments effectively reduced MDA levels, with high-dose AC restoring MDA to baseline values comparable to those in the NC group. Together, these findings demonstrate that AC attenuates Aβ accumulation in the hippocampus, ameliorates histopathological abnormalities, while reducing peripheral systemic oxidative stress as reflected by normalized serum MDA levels.

## 4 Discussion

Drug repurposing, the strategy of identifying novel therapeutic indications for already marketed medicines, has attracted growing attention as an accelerated developmental pipeline for neurodegenerative disease treatments (Cummings et al., 2025). This strategy carries particular value for disease areas where *de novo* drug discovery is prohibitively costly or where urgent medical needs remain unaddressed. AC, a marketed PDE5I, exhibits favorable pharmacokinetics profiles, rapid onset of action, and minimal side effects within its standard therapeutic dose range (Cui et al., 2021). Against this backdrop, the present work provides preliminary preclinical evidence supporting further exploration of AC for AD-relevant mechanisms.

PDE5, a cGMP-specific hydrolytic enzyme, has been identified as a potential target for neurological diseases and has been detected in the human brain (Ahmed et al., 2021). Multiple preclinical animal studies have verified that pharmacological PDE5 inhibition restores impaired cognitive function; among existing PDE5 inhibitors, sildenafil has already displayed robust anti-AD efficacy across several independent investigations (Sanders, 2020). In this study, *in silico* molecular docking and MD uncovered that aildenafil forms a far more stable binding complex within the PDE5 catalytic pocket relative to sildenafil, furnishing a clear structural mechanism underlying AC’s superior binding affinity. This structural advantage is functionally validated by our *in vitro* enzymatic assay, where aildenafil exerted potent PDE5A inhibitory activity with an IC50 of 0.66 nM (Table 2). While direct measurement of central brain cGMP levels remains a subject for future investigation, abundant published data confirm that PDE5 blockade modulates the cGMP/CREB signaling axis to rescue defective synaptic plasticity in AD animal models (Puzzo et al., 2009; Singh et al., 2024).

The APP/PS1 transgenic mouse model was selected for *in vivo* validation based on well-characterized pathological features recapitulating human AD (Shen et al., 2018). Excessive accumulation of Aβ peptides in AD is potentially driven by enhanced endoproteolytic cleavage of membrane-bound APP and/or overexpression of APP (Nunan and Small, 2000). Additionally, mutations in the *APP* and *PS1* genes have been found to mediate the Aβ accumulation by altering APP metabolism (Sasaguri et al., 2017). Therefore, APP/PS1 transgenic mice serve as a suitable model that recapitulates the main pathological features of AD. Our study demonstrates that AC improves spatial learning and memory, enhances locomotor activity, and reduces anxiety-related behaviors in APP/PS1 transgenic mice compared to untreated controls, with comparable therapeutic potency to the positive control memantine group. Improvements in pathological abnormalities within the hippocampal region were observed, including reduced Aβ aggregation, increased NEP expression. The significant mitigation of AD-associated abnormalities by AC supports the rationale for repurposing this drug as a therapeutic alternative to memantine.

Despite the encouraging multi-layered protective phenotypes observed in this study, several key limitations must be acknowledged. First, each experimental group used a sample size of n = 6. While this number falls within the commonly accepted range of 6–10 animals for APP/PS1 pathological and behavioral research, adheres to the 3R principles of animal ethics, and aligns with the exploratory, preliminary nature of our study, such a limited sample size precludes robust sex-stratified statistical analyses. Although male and female mice were evenly distributed across all groups, the mixed-sex design permits only a collective evaluation of therapeutic effects within a small cohort and does not allow for the analysis of sex-specific treatment differences. Second, the absence of pharmacokinetic and brain distribution data for AC precludes direct correlation between central exposure and the observed effects. Given divergent blood–brain barrier permeability across PDE5 inhibitors (Black et al., 2008; Mao et al., 2018), neurobiological outcomes reported for analog compounds cannot be reliably extrapolated to AC. Third, this work constitutes a phenotypic observational study rather than a targeted mechanistic exploration: we did not directly quantify core signaling intermediates of the proposed cGMP cascade, including intracellular cGMP abundance, PKG activation, or CREB phosphorylation status. While NEP upregulation provides a plausible intermediate linking PDE5 inhibition to diminished Aβ deposition, the causal linkage remains an open question requiring dedicated mechanistic investigation. The causal mechanistic chain connecting PDE5 blockade to the full spectrum of AC-mediated neuroprotective improvements remains correlative and requires dedicated mechanistic validation in subsequent assays.

In summary, this study comprehensively characterizes the multifaceted improvements induced by AC in the APP/PS1 mouse model of AD, encompassing behavioral, pathological, and oxidative stress endpoints. As a highly selective and potent PDE5 inhibitor, AC significantly mitigates learning and memory deficits, reduces Aβ accumulation, upregulates the Aβ-degrading enzyme NEP, and attenuates oxidative stress. Collectively, these findings deliver suggestive phenotypic data, indicating that AC repurposing merits further systematic evaluation as a prospective research direction for AD therapeutic development.

## Data Availability

The original contributions presented in the study are included in the article/Supplementary Material, further inquiries can be directed to the corresponding author.

## References

[B1] Abraham M. J. Murtola T. Schulz R. Páll S. Smith J. C. Hess B. (2015). GROMACS: high performance molecular simulations through multi-level parallelism from laptops to supercomputers. SoftwareX 1–2, 19–25. 10.1016/j.softx.2015.06.001 1

[B2] Ahmed W. S. Geethakumari A. M. Biswas K. H. (2021). Phosphodiesterase 5 (PDE5): structure-function regulation and therapeutic applications of inhibitors. Biomed. Pharmacother. 134, 111128. 10.1016/j.biopha.2020.111128 33348311

[B3] Alzheimer’s Association (2015). 2015 Alzheimer’s disease facts and figures. Alzheimer’s Dement. 11, 332–384. 10.1016/j.jalz.2015.02.003 25984581

[B4] Alzheimer’s Association (2025). Alzheimer’s disease facts and figures. Alzheimer’s Dement. 21, e70235. 10.1002/alz.70235 22404854

[B5] Atri A. (2019). Current and future treatments in Alzheimer’s disease. Semin. Neurol. 39, 227–240. 10.1055/s-0039-1678581 30925615

[B6] Avgerinos K. I. Manolopoulos A. Ferrucci L. Kapogiannis D. (2024). Critical assessment of anti-amyloid-β monoclonal antibodies effects in Alzheimer’s disease: a systematic review and meta-analysis highlighting target engagement and clinical meaningfulness. Sci. Rep. 14, 25741. 10.1038/s41598-024-75204-8 39468148 PMC11520896

[B7] Bernetti M. Bussi G. (2020). Pressure control using stochastic cell rescaling. J. Chem. Phys. 153, 114107. 10.1063/5.0020514 32962386

[B8] Black K. L. Yin D. Ong J. M. Hu J. Konda B. M. Wang X. (2008). PDE5 inhibitors enhance tumor permeability and efficacy of chemotherapy in a rat brain tumor model. Brain Res. 1230, 290–302. 10.1016/j.brainres.2008.06.122 18674521 PMC2632551

[B9] Bussi G. Donadio D. Parrinello M. (2007). Canonical sampling through velocity rescaling. J. Chem. Phys. 126, 014101. 10.1063/1.2408420 17212484

[B10] Case D. A. Aktulga H. M. Belfon K. Cerutti D. S. Cisneros G. A. Cruzeiro V. W. D. (2023). AmberTools. J. Chem. Inf. Model. 63, 6183–6191. 10.1021/acs.jcim.3c01153 37805934 PMC10598796

[B11] Cichero E. Calautti A. Francesconi V. Tonelli M. Schenone S. Fossa P. (2021). Probing *in silico* the benzimidazole privileged scaffold for the development of drug-like anti-RSV agents. Pharmaceuticals 14, 1307. 10.3390/ph14121307 34959708 PMC8707824

[B12] Cockerill G. S. Angell R. M. Bedernjak A. Chuckowree I. Fraser I. Gascon-Simorte J. (2021). Discovery of sisunatovir (RV521), an inhibitor of respiratory syncytial virus fusion. J. Med. Chem. 64, 3658–3676. 10.1021/acs.jmedchem.0c01882 33729773

[B13] Cui W.-S. Guan R.-L. Lei H.-E. Liu J.-H. Wang T. Zhu S.-N. (2021). Efficacy and safety of aildenafil citrate in Chinese men with erectile dysfunction: a multicenter, randomized, double-blind, placebo-controlled crossover trial. Transl. Androl. Urol. 10, 3358–3367. 10.21037/tau-21-441 34532260 PMC8421832

[B14] Cummings J. Osse A. M. L. Cammann D. Powell J. Chen J. (2024). Anti-amyloid monoclonal antibodies for the treatment of Alzheimer’s disease. BioDrugs 38, 5–22. 10.1007/s40259-023-00633-2 37955845 PMC10789674

[B15] Cummings J. L. Zhou Y. Van Stone A. Cammann D. Tonegawa-Kuji R. Fonseca J. (2025). Drug repurposing for Alzheimer’s disease and other neurodegenerative disorders. Nat. Commun. 16, 1755. 10.1038/s41467-025-56690-4 39971900 PMC11840136

[B16] Darden T. York D. Pedersen L. (1993). Particle mesh ewald: an *N* ⋅log(*N*) method for ewald sums in large systems. J. Chem. Phys. 98, 10089–10092. 10.1063/1.464397

[B17] Devan B. D. Bowker J. L. Duffy K. B. Bharati I. S. Jimenez M. Sierra-Mercado D. (2006). Phosphodiesterase inhibition by sildenafil citrate attenuates a maze learning impairment in rats induced by nitric oxide synthase inhibition. Psychopharmacology 183, 439–445. 10.1007/s00213-005-0232-z 16320087

[B18] Eberhardt J. Santos-Martins D. Tillack A. F. Forli S. (2021). AutoDock Vina 1.2.0: new docking methods, expanded force field, and Python bindings. J. Chem. Inf. Model. 61, 3891–3898. 10.1021/acs.jcim.1c00203 34278794 PMC10683950

[B19] Folch J. Busquets O. Ettcheto M. Sanchez-Lopez E. Castro-Torres R. D. Verdaguer E. (2018). Memantine for the treatment of dementia: a review on its current and future applications. J. Alzheimers Dis. 62, 1223–1240. 10.3233/JAD-170672 29254093 PMC5870028

[B20] García-Barroso C. Ricobaraza A. Pascual-Lucas M. Unceta N. Rico A. J. Goicolea M. A. (2013). Tadalafil crosses the blood–brain barrier and reverses cognitive dysfunction in a mouse model of AD. Neuropharmacology 64, 114–123. 10.1016/j.neuropharm.2012.06.052 22776546

[B21] Gasteiger J. Marsili M. (1980). Iterative partial equalization of orbital electronegativity—a rapid access to atomic charges. Tetrahedron 36, 3219–3228. 10.1016/0040-4020(80)80168-2

[B22] Hess B. Bekker H. Berendsen H. J. C. Fraaije J. G. E. M. (1997). LINCS: a linear constraint solver for molecular simulations. J. Comput. Chem. 18, 1463–1472. 10.1002/(SICI)1096-987X(199709)18:12<1463::AID-JCC4>3.0.CO;2-H

[B23] Hornak V. Abel R. Okur A. Strockbine B. Roitberg A. Simmerling C. (2006). Comparison of multiple Amber force fields and development of improved protein backbone parameters. Proteins 65, 712–725. 10.1002/prot.21123 16981200 PMC4805110

[B24] Jakalian A. Jack D. B. Bayly C. I. (2002). Fast, efficient generation of high‐quality atomic charges. AM1‐BCC model: II. Parameterization and validation. J. Comput. Chem. 23, 1623–1641. 10.1002/jcc.10128 12395429

[B25] Jehle A. Garaschuk O. (2022). The interplay between cGMP and calcium signaling in Alzheimer’s disease. IJMS 23, 7048. 10.3390/ijms23137048 35806059 PMC9266933

[B26] Jorgensen W. L. Chandrasekhar J. Madura J. D. Impey R. W. Klein M. L. (1983). Comparison of simple potential functions for simulating liquid water. J. Chem. Phys. 79, 926–935. 10.1063/1.445869

[B27] Kang B. W. Kim F. Cho J.-Y. Kim S. Rhee J. Choung J. J. (2022). Phosphodiesterase 5 inhibitor mirodenafil ameliorates Alzheimer-like pathology and symptoms by multimodal actions. Alz. Res. Ther. 14, 92. 10.1186/s13195-022-01034-3 35804462 PMC9264543

[B28] Lindorff‐Larsen K. Piana S. Palmo K. Maragakis P. Klepeis J. L. Dror R. O. (2010). Improved side‐chain torsion potentials for the Amber ff99SB protein force field. Proteins 78, 1950–1958. 10.1002/prot.22711 20408171 PMC2970904

[B29] Liu L. Xu H. Ding S. Wang D. Song G. Huang X. (2019). Phosphodiesterase 5 inhibitors as novel agents for the treatment of Alzheimer’s disease. Brain Res. Bull. 153, 223–231. 10.1016/j.brainresbull.2019.09.001 31493542

[B30] Mao F. Wang H. Ni W. Zheng X. Wang M. Bao K. (2018). Design, synthesis, and biological evaluation of orally available first-generation dual-target selective inhibitors of acetylcholinesterase (AChE) and phosphodiesterase 5 (PDE5) for the treatment of Alzheimer’s disease. ACS Chem. Neurosci. 9, 328–345. 10.1021/acschemneuro.7b00345 29068218

[B31] Nguyen H. V. Mital S. Knopman D. S. Alexander G. C. (2024). Cost-effectiveness of lecanemab for individuals with early-stage alzheimer disease. Neurology 102, e209218. 10.1212/WNL.0000000000209218 38484190

[B32] Nunan J. Small D. H. (2000). Regulation of APP cleavage by α-β- and γ-secretases. FEBS Lett. 483, 6–10. 10.1016/s0014-5793(00)02076-7 11033346

[B33] O’Boyle N. M. Banck M. James C. A. Morley C. Vandermeersch T. Hutchison G. R. (2011). Open babel: an open chemical toolbox. J. Cheminform 3, 33. 10.1186/1758-2946-3-33 21982300 PMC3198950

[B34] Pifarre P. Prado J. Baltrons M. A. Giralt M. Gabarro P. Feinstein D. L. (2011). Sildenafil (Viagra) ameliorates clinical symptoms and neuropathology in a mouse model of multiple sclerosis. Acta Neuropathol. 121, 499–508. 10.1007/s00401-010-0795-6 21234581

[B35] Puzzo D. Vitolo O. Trinchese F. Jacob J. P. Palmeri A. Arancio O. (2005). Amyloid-β peptide inhibits activation of the nitric Oxide/cGMP/cAMP-responsive element-binding protein pathway during hippocampal synaptic plasticity. J. Neurosci. 25, 6887–6897. 10.1523/JNEUROSCI.5291-04.2005 16033898 PMC6725343

[B36] Puzzo D. Staniszewski A. Deng S. X. Privitera L. Leznik E. Liu S. (2009). Phosphodiesterase 5 inhibition improves synaptic function, memory, and amyloid- load in an Alzheimer’s disease mouse model. J. Neurosci. 29, 8075–8086. 10.1523/JNEUROSCI.0864-09.2009 19553447 PMC6666028

[B37] Qiao O. Zhang X. Zhang Y. Ji H. Li Z. Han X. (2021). Cerebralcare Granule® enhances memantine hydrochloride efficacy in APP/PS1 mice by ameliorating amyloid pathology and cognitive functions. Chin. Med. 16, 47. 10.1186/s13020-021-00456-9 34183022 PMC8240384

[B38] Reitz C. Mayeux R. (2014). Alzheimer disease: epidemiology, diagnostic criteria, risk factors and biomarkers. Biochem. Pharmacol. 88, 640–651. 10.1016/j.bcp.2013.12.024 24398425 PMC3992261

[B39] Ross E. L. Weinberg M. S. Arnold S. E. (2022). Cost-effectiveness of aducanumab and donanemab for early alzheimer disease in the US. JAMA Neurol. 79, 478. 10.1001/jamaneurol.2022.0315 35344024 PMC8961406

[B40] Roymans D. Alnajjar S. S. Battles M. B. Sitthicharoenchai P. Furmanova-Hollenstein P. Rigaux P. (2017). Therapeutic efficacy of a respiratory syncytial virus fusion inhibitor. Nat. Commun. 8, 167. 10.1038/s41467-017-00170-x 28761099 PMC5537225

[B41] Sanders O. (2020). Sildenafil for the treatment of Alzheimer’s disease: a systematic review. J. Alzheimer's Dis. Rep. 4, 91–106. 10.3233/ADR-200166 32467879 PMC7242821

[B42] Sasaguri H. Nilsson P. Hashimoto S. Nagata K. Saito T. De Strooper B. (2017). APP mouse models for Alzheimer’s disease preclinical studies. EMBO J. 36, 2473–2487. 10.15252/embj.201797397 28768718 PMC5579350

[B43] Scheltens P. De Strooper B. Kivipelto M. Holstege H. Chételat G. Teunissen C. E. (2021). Alzheimer’s disease. Lancet 397, 1577–1590. 10.1016/S0140-6736(20)32205-4 33667416 PMC8354300

[B44] Shen Z. Lei J. Li X. Wang Z. Bao X. Wang R. (2018). Multifaceted assessment of the APP/PS1 mouse model for Alzheimer’s disease: applying MRS, DTI, and ASL. Brain Res. 1698, 114–120. 10.1016/j.brainres.2018.08.001 30077647

[B45] Shuvaev A. N. Belozor O. S. Mozhei O. I. Mileiko A. G. Mosina L. D. Laletina I. V. (2022). Memantine disrupts motor coordination through anxiety-like behavior in CD1 mice. Brain Sci. 12, 495. 10.3390/brainsci12040495 35448027 PMC9027563

[B46] Singh N. K. Singh P. Varshney P. Singh A. Bhushan B. (2024). Multimodal action of phosphodiesterase 5 inhibitors against neurodegenerative disorders: an update review. J. Biochem. Mol. Tox. 38, e70021. 10.1002/jbt.70021 39425458

[B47] Stepanyan T. D. Farook J. M. Kowalski A. Kaplan E. Barron S. Littleton J. M. (2008). Alcohol withdrawal‐induced hippocampal neurotoxicity *in vitro* and seizures *in vivo* are both reduced by memantine. Alcohol. Clin. Exp. Res. 32, 2128–2135. 10.1111/j.1530-0277.2008.00801.x 18828800

[B48] Tropea M. R. Gulisano W. Vacanti V. Arancio O. Puzzo D. Palmeri A. (2022). Nitric oxide/cGMP/CREB pathway and amyloid-beta crosstalk: from physiology to Alzheimer’s disease. Free Radic. Biol. Med. 193, 657–668. 10.1016/j.freeradbiomed.2022.11.022 36400326

[B49] Trott O. Olson A. J. (2010). AutoDock vina: improving the speed and accuracy of docking with a new scoring function, efficient optimization, and multithreading. J. Comput. Chem. 31, 455–461. 10.1002/jcc.21334 19499576 PMC3041641

[B50] Uppuluri C. T. Prasad Y. S. K. V. Asadi K. Kumari T. P. Pelluri R. Chakravarthi G. (2026). PDE5 inhibitors as modulators of Alzheimer’s-associated inflammation and oxidative stress: a meta-analytical assessment of preclinical studies. Mol. Neurobiol. 63, 322. 10.1007/s12035-025-05635-5 41433006

[B51] Valdés-Tresanco M. S. Valdés-Tresanco M. E. Valiente P. A. Moreno E. (2021). gmx_MMPBSA: a new tool to perform end-state free energy calculations with GROMACS. J. Chem. Theory Comput. 17, 6281–6291. 10.1021/acs.jctc.1c00645 34586825

[B52] Van Dam D. Abramowski D. Staufenbiel M. De Deyn P. P. (2005). Symptomatic effect of donepezil, rivastigmine, galantamine and memantine on cognitive deficits in the APP23 model. Psychopharmacology 180, 177–190. 10.1007/s00213-004-2132-z 15654502

[B53] Vendeville S. Tahri A. Hu L. Demin S. Cooymans L. Vos A. (2020). Discovery of 3-({5-Chloro-1-[3-(methylsulfonyl)propyl]-1 *H* -indol-2-yl}methyl)-1-(2,2,2-trifluoroethyl)-1,3-dihydro-2 *H* -imidazo[4,5- *c* ]pyridin-2-one (JNJ-53718678), a Potent and Orally Bioavailable Fusion Inhibitor of Respiratory Syncytial Virus. J. Med. Chem. 63, 8046–8058. 10.1021/acs.jmedchem.0c00226 32407115

[B54] Verma A. Kumar Waiker D. Bhardwaj B. Saraf P. Shrivastava S. K. (2022). The molecular mechanism, targets, and novel molecules in the treatment of Alzheimer’s disease. Bioorg. Chem. 119, 105562. 10.1016/j.bioorg.2021.105562 34952243

[B55] Wang J. Wolf R. M. Caldwell J. W. Kollman P. A. Case D. A. (2004). Development and testing of a general amber force field. J. Comput. Chem. 25, 1157–1174. 10.1002/jcc.20035 15116359

[B69] Walsh R. N. Cummins R. A. (1976). The Open-Field Test: a critical review. Psychol. Bull. 83, 482–504. 10.1037/0033-2909.83.3.482 17582919

[B56] Weeks J. L. Blount M. A. Beasley A. Zoraghi R. Thomas M. K. Sekhar K. R. (2005). Radiolabeled ligand binding to the catalytic or allosteric sites of PDE5 and PDE11. Methods Mol. Biol. 307, 239–262. 10.1385/1-59259-839-0:239 15988068

[B57] Wu D. Zhang T. Chen Y. Huang Y. Geng H. Yu Y. (2017). Discovery and optimization of Chromeno[2,3- *c* ]pyrrol-9(2 *H*)-ones as novel selective and orally bioavailable phosphodiesterase 5 inhibitors for the treatment of pulmonary arterial hypertension. J. Med. Chem. 60, 6622–6637. 10.1021/acs.jmedchem.7b00523 28686445

[B58] Wu Y. Li Z. Huang Y.-Y. Wu D. Luo H.-B. (2018). Novel phosphodiesterase inhibitors for cognitive improvement in Alzheimer’s disease: miniperspective. J. Med. Chem. 61, 5467–5483. 10.1021/acs.jmedchem.7b01370 29363967

[B59] Xiong X. Lv G. King J. J. Li M. Yuan J. Lu Z. K. (2025). Cost-effectiveness analysis of aducanumab versus placebo for patients with mild cognitive impairment and mild Alzheimer’s disease. BMJ Open 15, e090403. 10.1136/bmjopen-2024-090403 40250880 PMC12007052

[B60] Yu D.-T. Li R.-X. Sun J.-R. Rong X.-W. Guo X.-G. Zhu G.-D. (2025). Global mortality, prevalence and disability-adjusted life years of Alzheimer’s disease and other dementias in adults aged 60 years or older, and the impact of the COVID-19 pandemic: a comprehensive analysis for the global burden of disease 2021. BMC Psychiatry 25, 503. 10.1186/s12888-025-06661-2 40389943 PMC12090430

[B61] Zhang J. Guo J. Zhao X. Chen Z. Wang G. Liu A. (2013). Phosphodiesterase-5 inhibitor sildenafil prevents neuroinflammation, lowers beta-amyloid levels and improves cognitive performance in APP/PS1 transgenic mice. Behav. Brain Res. 250, 230–237. 10.1016/j.bbr.2013.05.017 23685322

[B62] Zhang R. Zhang Y. Zheng W. Shang W. Wu Y. Li N. (2022). Oral remdesivir derivative VV116 is a potent inhibitor of respiratory syncytial virus with efficacy in mouse model. Sig. Transduct. Target. Ther. 7, 123. 10.1038/s41392-022-00963-7 35429988 PMC9012943

[B63] Zhang J. Zhang Y. Wang J. Xia Y. Zhang J. Chen L. (2024). Recent advances in Alzheimer’s disease: mechanisms, clinical trials and new drug development strategies. Sig. Transduct. Target. Ther. 9, 211. 10.1038/s41392-024-01911-3 PMC11344989 39174535

[B64] Zhao Y. Zhao B. (2013). Oxidative stress and the pathogenesis of Alzheimer’s disease. Oxid. Med. Cell. Longev. 2013, 1–10. 10.1155/2013/316523 23983897 PMC3745981

[B65] Zheng Q. Wang X. (2025). Alzheimer’s disease: insights into pathology, molecular mechanisms, and therapy. Protein Cell 16, 83–120. 10.1093/procel/pwae026 38733347 PMC11786724

[B66] Zhu L. Yang J. Xue X. Dong Y. Liu Y. Miao F. (2015). A novel phosphodiesterase-5 inhibitor: yonkenafil modulates neurogenesis, gliosis to improve cognitive function and ameliorates amyloid burden in an APP/PS1 transgenic mice model. Mech. Ageing Dev. 150, 34–45. 10.1016/j.mad.2015.07.002 26200391

[B67] Zhu Q. McLellan J. S. Kallewaard N. L. Ulbrandt N. D. Palaszynski S. Zhang J. (2017). A highly potent extended half-life antibody as a potential RSV vaccine surrogate for all infants. Sci. Transl. Med. 9, eaaj1928. 10.1126/scitranslmed.aaj1928 28469033

[B68] Zuccarello E. Acquarone E. Calcagno E. Argyrousi E. K. Deng S.-X. Landry D. W. (2020). Development of novel phosphodiesterase 5 inhibitors for the therapy of Alzheimer’s disease. Biochem. Pharmacol. 176, 113818. 10.1016/j.bcp.2020.113818 31978378 PMC7263960

