# Supplementary material for: Aildenafil citrate with superior PDE5 selectivity ameliorates cognitive and behavioral impairments in APP/PS1 Alzheimer’s disease mice: a preclinical phenotypic study
Source: Front Pharmacol. 2026 Sep 16;17:1933588. doi: 10.3389/fphar.2026.1933588 (PMC13624397; doi:10.3389/fphar.2026.1933588)
Supplement: Supplementary file 1 [file Supplementaryfile1.docx]

***Supplementary Material***

# Aildenafil citrate with superior PDE5 selectivity ameliorates cognitive and behavioral impairments in APP/PS1 Alzheimer's disease mice: a preclinical phenotypic study

**Shaowei Han^1,2,†^, Fang Liu^1,2,†^, Dawei Wang^1^, Gengshen Song^1,2,^***

^1^Beijing Youcare Kechuang Pharmaceutical Technology Co., Ltd., Beijing, China;

^2^ Hangzhou Tianlong Pharmaceutical Co., Ltd., Zhejiang, China.

† These authors contributed equally to this work and share first authorship

* Correspondence: songgengshen@youcareyk.com

**Supplementary Scheme S1. Synthesis of AC.** Reagents and conditions: (i) 2-ethoxy benzoic acid, ClSO_3_H, SOCl_2,_ 5-chlorosulphonyl-2-ethoxy benzoic acid, < 25°C to rt, 18 h; (ii) 6-dimethylpiperazine, 2-ethoxy-5-(cis-2,6-dimethypiperazin-4-ylsulphonyl) benzoic acid, 10 °C, 2 h; (iii) SOCl_2_, 2-ethoxy-5-(cis-2,6-dimethylpiperazin-4-ylsulphonyl) benzoyl chloride, reflux, 3 h; (iv) 1-methyl-4-amino-3-n-propyl pyrazole-5-formamide, 4-dimethylaminopyridine, triethylamine, < 10 °C, 2 h; (v) metallic potassium, tert-butyl alcohol, 5-[[2-ethoxy-5-(cis-2,6-dimethylpiperazin-4-ylsulphonyl)phenyl]]-1-methyl-3-npropyl-7,6-dihydro-1H-pyrazolo[4,3-d]pyri midin-7- one, reflux, 8 h.

**Supplementary Figure S1.** ¹H-NMR spectrum of AC.

**Supplementary Figure S2.** ^13^C-NMR spectrum of AC.

**Supplementary Figure S3.** Escape latency in APP/PS1 transgenic mice or WT controls was evaluated by Barnes maze test prior to treatment. Data were presented as mean ± SD (n = 6 each group). Statistical differences were determined using one-way ANOVA, n.s. means no significance.

**Supplementary Figure S4.** Percentage of distance traveled in the central zone. Data were presented as mean ± SD (n = 6 each group).

**Supplementary Figure S5.** Immunohistochemical analysis of neprilysin (NEP). Data were presented as mean ± SD (n = 6 each group).
